# Uveitis- a rare disease often associated with systemic diseases and infections- a systematic review of 2619 patients

**DOI:** 10.1186/1750-1172-7-57

**Published:** 2012-08-29

**Authors:** Talin Barisani-Asenbauer, Saskia M Maca, Lamiss Mejdoubi, Wolfgang Emminger, Klaus Machold, Herbert Auer

**Affiliations:** 1 Department of Ophthalmology and Optometry, Medical University Vienna, Waehringer Guertel 18-20, A-1090, Vienna, Austria; 2 Laura Bassi Centre for Ocular Inflammation and Infection, Institute for Specific Prophylaxis and Tropical Medicine, Medical University Vienna, Kinderspitalgasse 15, A-1090, Vienna, Austria; 3 Department of Ophthalmology, Hietzing Hospital, Wolkersbergenstrasse 1, A-1130, Vienna, Austria; 4 Department of Pediatrics and adolescent medicine. Division of Pediatric Nephrology and Gastroenterology, Medical University Vienna, Waehringer Guertel 18-20, A-1090, Vienna, Austria; 5 Department of Rheumatology, Medical University Vienna, Waehringer Guertel 18-20, A-1090, Vienna, Austria; 6 Department of Medical Parasitology, Institute for Specific Prophylaxis and Tropical Medicine, Medical University Vienna, Waehringer Guertel 18-20, A-1090, Vienna, Austria

**Keywords:** Uveitis, Etiology, Systemic associations, Arthritis, Infections

## Abstract

**Background:**

Uveitis is an autoimmune disease of the eye that refers to any of a number of intraocular inflammatory conditions. Because it is a rare disease, uveitis is often overlooked, and the possible associations between uveitis and extra-ocular disease manifestations are not well known. The aim of this study was to characterize uveitis in a large sample of patients and to evaluate the relationship between uveitis and systemic diseases.

**Methods:**

The present study is a cross-sectional study of a cohort of patients with uveitis. Records from consecutive uveitis patients who were seen by the Uveitis Service in the Department of Ophthalmology at the Medical University of Vienna between 1995 and 2009 were selected from the clinical databases. The cases were classified according to the Standardization of Uveitis Nomenclature Study Group criteria for Uveitis.

**Results:**

Data were available for 2619 patients, of whom 59.9% suffered from anterior, 14.8% from intermediate, 18.3% from posterior and 7.0% from panuveitis. 37.2% of all cases showed an association between uveitis and extra-organ diseases; diseases with primarily arthritic manifestations were seen in 10.1% of all cases, non-infectious systemic diseases (i.e., Behçet´s disease, sarcoidosis or multiple sclerosis) in 8.4% and infectious uveitis in 18.7%. 49.4% of subjects suffering from anterior uveitis tested positively for the HLA-B27 antigen. In posterior uveitis cases 29% were caused by ocular toxoplasmosis and 17.7% by multifocal choroiditis.

**Conclusion:**

Ophthalmologists, rheumatologists, infectiologists, neurologists and general practitioners should be familiar with the differential diagnosis of uveitis. A better interdisciplinary approach could help in tailoring of the work-up, earlier diagnosis of co-existing diseases and management of uveitis patients.

## Background

Uveitis is a sight-threatening inflammation inside the eye that affects both the uveal tract (which is composed of the iris, choroid, and ciliary body and which is the blood-supplying layer inside of the eye), and adjacent structures (including the sclera, cornea, vitreous humor, retina and optic nerve head). Because the disease involves recurrent intraocular inflammation, uveitis can cause transient or permanent visual impairment and ocular complications that are not responsive to therapy [1-4]. Uveitis can occur either as a co-manifestation of various autoimmune disorders and infections or as a side effect of medications and toxins, or it can arise as a purely idiopathic ocular inflammation [3,5-10].

The prevalence of uveitis is estimated at 38 cases per 100,000 people, so it meets the criteria for classification as a rare disease [2,11-19]. It is particularly prevalent in younger people; the mean age of uveitis patients at the onset of the disease is less than 40 years of age [20-22].

Although it is an orphan disease, uveitis is the fourth most common cause of blindness among the working-age population in the developed world, and its economical and social impact not yet been evaluated [3,20-22].

The referral of a patient to a uveitis expert is often delayed because uveitis is commonly unknown, and it is therefore under-recognized. This delay in diagnosis and referral increases the risk that uveitis will result in irreversible damage to various ocular structures. Prompted by the hypothesis that highlighting systemic associations would optimize the referral of patients with uveitis, we conducted this systematic review to analyze the distributions of uveitis subtypes and their extra-organ manifestations.

## Patients and methods

The institutional review board of the Medical University of Vienna approved this study. Since September 1995, data from all patients referred to the Uveitis Unit of the Department of Ophthalmology, Medical University of Vienna, were systematically recorded. A total of 2619 consecutive patient records were analyzed for this study.

The uveitis subtypes were classified based on the specific disease patterns and diagnoses following the recommendations of the International Uveitis Study Group and the Standardization of Uveitis Nomenclature (SUN) working group [23,24]. Guided by the medical history of each patient, investigations of the anatomical location and character of the inflammation as well as subsequent diagnostics were performed using a tailored approach [25,26]. All patients were treated in a multidisciplinary setting and referred to the respective specialist if systemic disease was suspected. Factors considered to satisfy the criteria of specific diagnosis included well-defined a etiology, typical clinical appearance and history or classification based on pathological or pathognomonic laboratory parameters.

All patients with acute anterior uveitis were typed selectively for presence of the HLA-B27 antigen, as HLA-B27–associated acute anterior uveitis with or without systemic disease is recognized as a specific uveitis entity [27]. All uveitis patients with multifocal choroiditis showing the typical clinical appearance of birdshot choroidopathy were typed for the HLA-A29 antigen [28]. These two HLA-antigen predispositions are considered to be pathognomonic when combined with the typical clinical appearance.

Diseases belonging to the white dot syndromes which will be discussed in this article are acute posterior multifocal placoid pigment epitheliopathy (APMPPE), multiple evanescent white dot syndrome (MEWDS), birdshot retinochoroidopathy (BSRC), multifocal choroiditis (MFC), punctuate inner choroidopathy (PIC), acute zonal occult outer retinopathy (AZOOR) and serpiginous choroiditis.

Descriptive statistics were computed using SPSS 17.0. Values are given as ranges, percentages or means ± standard error of the mean, as applicable.

## Results

The patient sample comprised 1339 women and 1278 men (51.1% and 48.2%, respectively). Uveitis occurred at a mean age of 38.8 ± 18.3 years (range 1-87). The age at the onset of the disease was between 17-60 years in 79.8% of the patients. 9.2% of all cases were children (<17 years) and 11% were elderly patients (>60 years).

**Figure 1:**
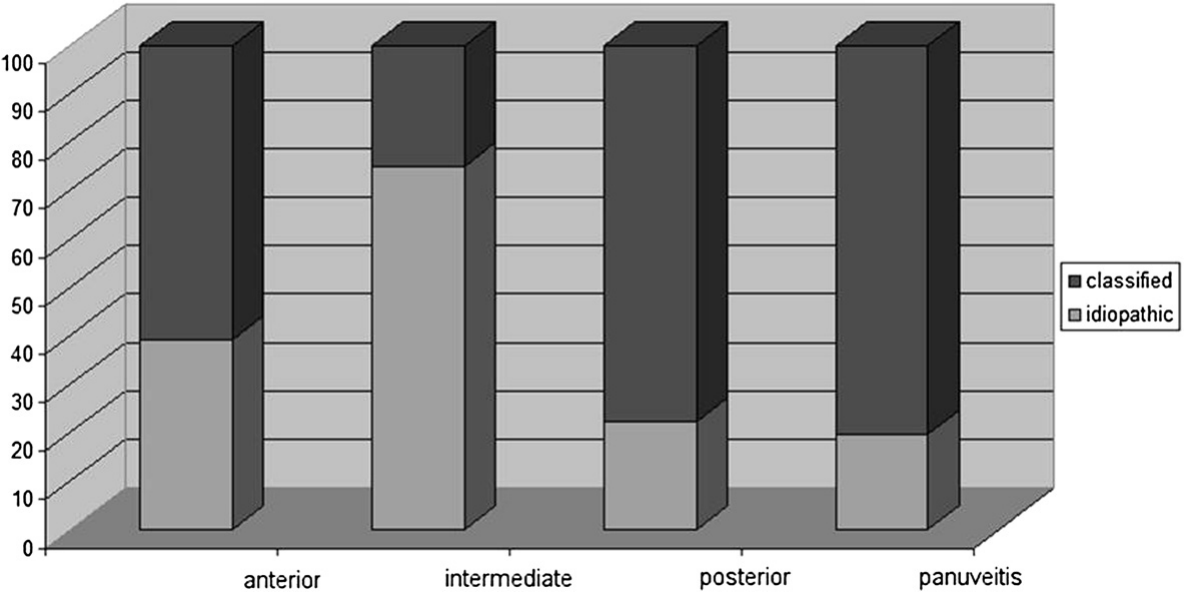
Percentage of unclassified and classified cases according to anatomical localization of uveitis.

The anatomical classification of uveitis was anterior in 59.9%, intermediate in 14.8%, posterior in 18.3% and panuveitis in 7% of cases. Figure 1 summarizes the distribution of unclassified and classified cases in these four groups.

### Specific diagnoses

Diseases with primarily arthritic manifestations and uveitis were diagnosed in 265 cases (10.1%). Of these, the specific a etiologies included enclosing spondylitis (n = 143), rheumatoid arthritis (n = 42), juvenile idiopathic arthritis (JIA) (n = 58), reactive arthritis (n = 5) and psoriatic arthritis (n = 17).

Infectious uveitis was diagnosed in 495 patients (19.0%). Diagnoses included 226 viral [ocular herpes virus reactivation (n = 201), systemic varicella virus infection (VZV) (n = 14), systemic cytomegaly virus infection (CMV) (n = 5), other (n = 10)], 198 parasitic [toxplasomosis (n = 173), 18 toxocarosis (n = 18), other (n = 7)], 47 bacterial and 24 fungal infections. Of these cases, 99 were associated with current systemic infections (Table 1).

**Table 1:** Current systemic infections agents as causes of active uveitis

**Viral (n = 21)**	**Bacterial (n = 47)**	**Parasites (n = 7)**	**Fungal (n = 24)**
VZV (n = 14)	*Mycobacterium tuberculosis* (n = 21)	*Ascaris lumbricoides* (n = 2)	*Candida albicans* (n = 22)
CMV (n=5)	*Chlamydia trachomatis* (n = 10)	Filaria (n=2)	*Aspergillus fumigatus* (n = 2)
Other (n=2)	*Treponema pallidum* (n = 9)	*Giarda lamblia* (n = 2)	
	*Borrelia Burgdorferi* (n = 7)	*Plasmodium falciparum* (n = 1)	

*VZV*: varicella virus infection; *CMV*: cytomegaly virus infection.

Of the purely ocular infections, 187 cases were diagnosed with herpetic anterior uveitis, 14 with acute retinal necrosis (ARN), 173 with ocular toxoplasmosis and 18 with ocular toxocarosis.

Non-infectious systemic diseases with primarily non-arthritic manifestations were diagnosed in 221 cases (8.4%). These included Behçet´s disease (n = 49), sarcoidosis (n = 64), Crohn´s disease (n = 30), multiple sclerosis (MS) (n = 25), ulcerative colitis (n = 14), Whipple´s disease (n = 2), Vogt-Koyanagi-Harada disease (VKH) (n = 11), systemic lupus erythematosus (SLE) (n = 7) and masquerade syndromes (MASQ) (n = 19).

Ocular syndromes such as HLA-B27–associated acute anterior uveitis without systemic disease (HLA-B27 AAU) (n = 271), Fuchs’ heterochromic cyclitis (HCF) (n = 88), serpiginous choroidopathy (n = 23), multifocal choroiditis (n = 86), birdshot choroidopathy (n = 10), acute posterior multifocal placoid pigment epitheliopathy (APMPPE) (n = 8), multiple evanescent white dot syndrome (n = 6), glaucomatous cyclonic crisis (n = 10) and sympathetic ophthalmia (n = 2), were found in 503 (19.2%) cases.

### Anatomical location and specific entities

In the anterior uveitis (AU) group, specific diagnoses could be established in 60.9% of cases. HLA-B27–associated acute anterior uveitis was diagnosed in 414 cases (30.5% of AU). These cases were either related to underlying HLA-B27–associated systemic diseases such as enclosing spondylitis (n = 143) or to purely ocular disease (271 cases).

Herpetic anterior uveitis and HCF were found in 11.4% and 4.5% of cases, respectively. JIA was seen in 3.4% of AU patients. In addition, uveitis was associated with sarcoidosis in 2.1% of cases, and Behçet´s disease in 0.9%. Systemic infections, MASQ, glaucomatous cyclonic crisis, and ulcerative colitis were each seen in less than 1% of AU patients.

75% of patients with intermediate uveitis remained without specific diagnosis. 51.5% of all IU patients were diagnosed with pars planets; 4.9% of these cases were associated with MS, 3.6% with HCF with dense vitreous opacities, 1.5% with sarcoidosis and <1% with scleroderma or toxocara canis.

In the posterior uveitis group, a specific diagnosis could be established in 78.1% of patients. Ocular toxoplasmosis was present in 29%, followed by multifocal choroiditis in 17.7% and serpiginous choroidopathy in 4.8%. Posterior uveitis was associated with systemic infections in 5.8%, sarcoidosis in 2.5%, VKH in 1.8%, ocular toxocarosis in 1.4% and Behçet´s disease in 2.5% of cases. ARN, MASQ, Whipple's disease and Crohn’s disease were each diagnosed in less than 1% of posterior Uveitis patients.

For patients with panuveitis, a specific diagnosis could be established in 80.4% of cases. Panuveitis was associated with systemic infections in 26.7%, ocular toxoplasmosis in 15.8%, sarcoidosis in 7.1%, Behçet's disease in 11.4%, VKH in 3% and acute retinal necrosis in 4.3% of cases.

Of the 2619 Uveitis cases, 39.4% stayed unclassified regarding specific diagnoses. The most predominant specific entity was HLA-B27–associated anterior uveitis (HLA-B27 AAU, with or without systemic disease), which was found in 19.5% of all patients, followed by ocular toxoplasmosis in 6.6%, herpetic uveitis in 7.1%, white dot syndromes (WDS) in 5.5%, systemic infections in 4%, HCF in 3.4%, sarcoidosis in 2.4%, Behçet’s disease in 1.9%, JIA in 2.2% and rheumatoid arthritis in 1.6% of the patients. Table 2 summarizes the most common etiologies of uveitis cases and their anatomical distribution.

**Table 2:** Etiology of endogenous uveitis cases

	**SUN-localisation**				**Total**
	**Anterior**	**Intermediate**	**Posterior**	**Panuveitis**	
Unclassified	**598**	**290**	**105**	**36**	**1029**
HLA-B27 AAU	**480**				**480**
Viral infections	**183**	**9**	**17**	**17**	**226**
Parasitic infections	**7**	**5**	**153**	**33**	**198**
Ocular syndromes	**79**	**15**	**142**	**14**	**250**
Sarcoidosis	**34**	**7**	**10**	**13**	**64**
JIA	**52**	**1**	**0**	**5**	**58**
Behçet’s disease	**14**	**2**	**12**	**21**	**49**
Other autoimmune diseases	**31**	**7**	**5**	**5**	**48**
Bacterial infections	**14**	**10**	**11**	**12**	**47**
Rheumatoid arthritis	**36**	**5**	**0**	**0**	**41**
MS	**4**	**19**	**2**	**0**	**25**
Fungal infections	**2**	**3**	**7**	**12**	**24**
MASQ	**7**	**4**	**5**	**4**	**20**

*JIA*: juvenile idiopathic arthritis; *MS*: multiple sclerosis; *MASQ*: masquerade syndromes.

### Age at onset of disease

**Figure 2:**
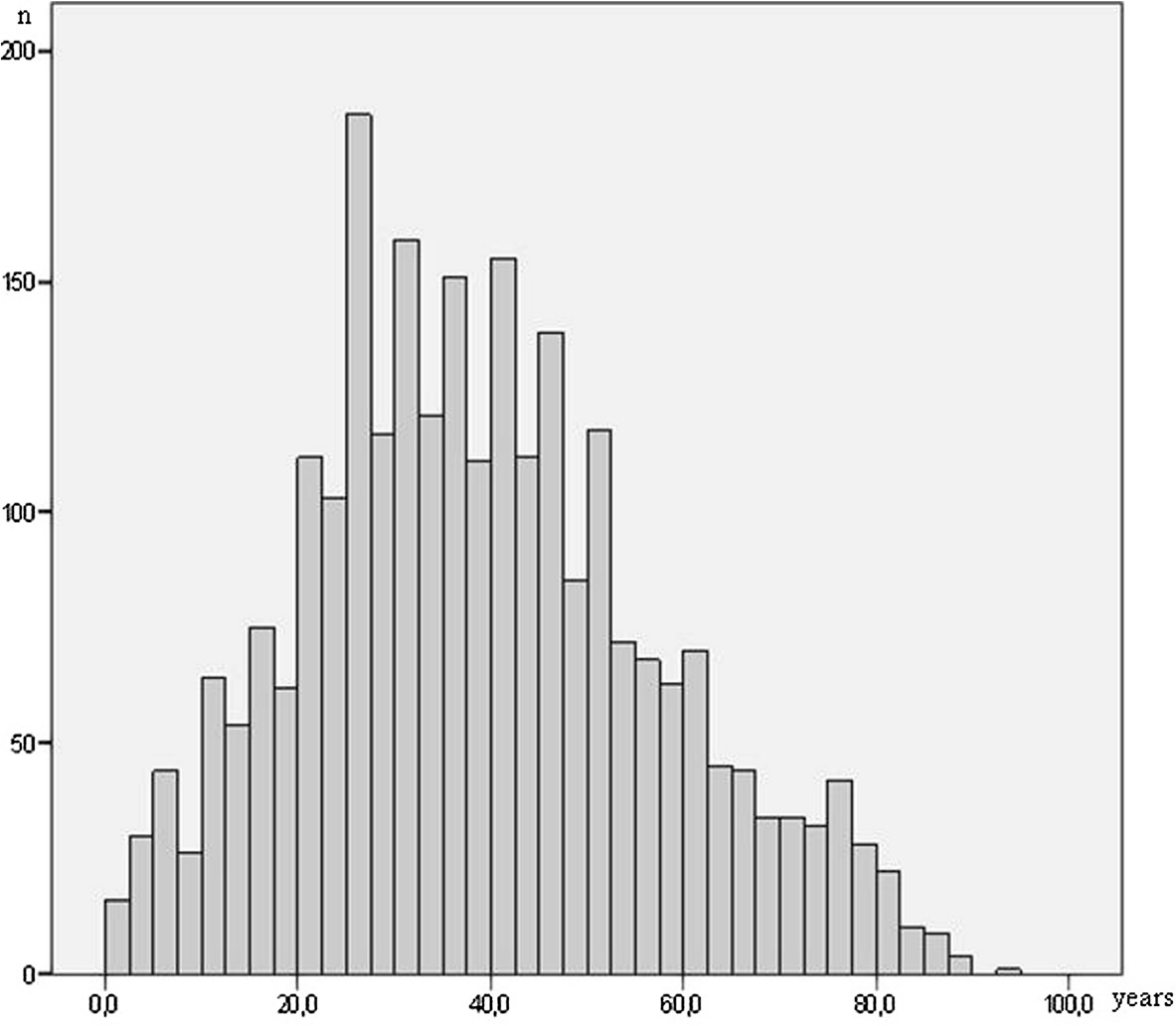
Distribution of patients’ age at disease onset.

The distribution of the patients’ age at disease onset is shown in Figure 2. The mean age at the onset of disease for the different specific entities can be seen in Table 3.

**Table 3:** Specific entities found in at least 5 patients and age at onset of disease

	**Mean**	**Min**	**Max**	**Main (yrs)**
JIA	10.8	1	29	<17
APMPPE	20.2	17	27	17-40
Reactive arthritis	34.7	7	66	>17
SLE	30.7	13	53	36-60
Toxoplamosis	29.2	1	79	17-40
Crohn´s disease	34.7	18	64	17-40
Reiter´s disease	35.5	13	64	17-35
Ulcerative colitis	43.4	24	49	17-60
HCF	32.4	12	70	<40
Behcet´s disease	31.1	15	55	<40
Ocular toxocarosis	47.2	11	71	>17
HLA-B27 only eye	37.8	9	82	17-60
Unclassified cases	39.5	2	87	17-60
Psoriatric arthritis	39.8	23	49	36-60
Ancyl. spondylitis	39.5	17	75	17-60
MFC	38.6	16	77	17-60
MS	35.9	14	77	17-40
Sarcoidosis	40.1	5	78	17-60
Serpiginous	45.3	15	76	>36
ARN	43.9	7	62	1 > 17
Ocular herpes	47.8	2	88	>36
Systemic infections	47.5	3	80	17-60
VKH	42.6	30	78	>30
Birdshot	51.4	32	75	36-60
MASQ	55.2	10	80	>36

For each classification, mean age of onset of disease (mean), minimum age at onset of disease (min), maximum age at onset of disease (max) and the main age group where this specific entity is found (main).

*JIA*: juvenile idiopathic arthritis; *APMPPE*: acute posterior multifocal placoid pigment epitheliopathy; *SLE*: systemic lupus erythematosus; *HCF*: Fuchs’ heterochromic cyclitis; *MFC*: multifocal choroiditis; *MS*: multiple sclerosis; *ARN*: acute retinal necrosis; *VKH*: Vogt-Koyanagi-Harada disease; *MASQ*: masquerade syndromes.

In the <7 years age group (n = 91, 3.45% of all patients), anterior uveitis represented the predominant anatomic group. The spectrum of specific diagnoses was narrow; JIA (29.6%), pars planets (10.3%), ocular toxoplasmosis (9.9%), sarcoidosis (2.2%), ocular herpes infection (6.6%), reactive arthritis (2.6%) and systemic infection (3.3%) were found, 35.1% of cases remained unclassified. In children between the ages of 7 and 17 years at the onset of disease (n = 215), an increase in the proportion of intermediate uveitis was noted (35.3%). Specific diagnoses were established in 53.9% of the cases. These included JIA (10.6%), ocular toxoplasmosis (14.4%), ocular herpes infection (3.7%), HLA-B27+ AAU (3.7%), HCF (4.6%), Behçet’s disease (1.8%), ocular toxocarosis (0.9%), systemic infections (1.7%) and sarcoidosis (3.2%), with serpiginous choroiditis, multifocal choroiditis, SLE and chronic polychondritis each observed in less than 1% of patients.

In two-thirds of the patients, disease onset occurred between 17 and 60 years of age. In this age group, the disease entities were diverse and are listed in Table 2.

Onset of disease after the age of 61 years was found in 13.5% of the patients; the largest group had anterior uveitis (63.1%). The diversity of specific entities was again reduced; 44.7% of patients were without specific diagnoses, 17.5% had ocular herpes infections, 6.2% had systemic infections, 8.4% had HLA-B27+ AAU, 2.8% had rheumatoid arthritis, 2.5% had ocular toxoplasmosis, 2.5% had WDS, 1.4% had serpiginous choroiditis, 2.2% had ocular toxocarosis, 1.9% had sarcoidosis and <1% had either multifocal choroiditis, birdshot chorioretinopathy or reactive arthritis disease. There were no cases of Behçet´s disease and just one case of MS in the group with onset ages of over 60 years.

## Discussion

In this study, we were able to establish specific diagnoses in 60.6% of 2619 patients; this is consistent with previous studies that reported ranges between 47 and 75% [29]. The relative frequencies of anatomical classifications are comparable to data published in other Middle European series from tertiary care centers but differ from the data of other Northern European countries, where up to 96% of the cases were reported to have anterior uveitis [30-35]. In the posterior and panuveitis groups, the rate of unclassified cases was the lowest at around 20%, and in the posterior uveitis group the frequency of toxoplasmosis was even higher than that among unclassified cases.

Overall, the largest diagnostic group comprised patients with ocular syndromes, followed by those with infectious diseases, arthritic diseases and, lastly, non-infectious systemic diseases.

When considering all patients, the major specific entities were HLA-B27 + AAU with or without systemic disease (19.5%), herpetic uveitis (7.1%) and ocular toxoplasmosis (6.6%).

The differential diagnosis of uveitis has changed over time. Tuberculosis and syphilis, the former main causes of uveitis, are now diagnosed in only 2.4% of patients [36,37]. More recently, however, increased frequency of these diseases has been noted. Factors that affect the changing patterns of uveitis include the rise of autoimmune diseases, appearance of new infections, description of new disease entities, better treatment of certain diseases, availability of new diagnostic tests and more refined classification of uveitis cases.

In the present study, the frequencies of different uveitis entities in different age groups were analyzed. In small children, only a few entities were discerned, including JIA, ocular toxoplasmosis, herpetic uveitis, pars planets, sarcoidosis, reactive arthritis, systemic infections and idiopathic anterior uveitis. With increasing age, the diversity of possible entities grew, reaching a peak between 30–40 years. In the elderly, the spectrum of specific diagnoses was again narrower; the major specific entities consisted of infections (herpes virus and toxoplasmosis) and masquerade syndromes. Some uveitis entities such as HCF, Behçet´s disease, and APMPPE appeared to affect adolescents or young adults with the greatest frequency; in children, JIA-associated Uveitis was most frequent. Nevertheless, most entities were observed in all age groups.

The high percentage of uveitis patients with systemic diseases and infections underpins the necessity of an interdisciplinary approach to uveitis therapy. Furthermore, rheumatologists and ophthalmologists have to be aware that a plethora of systemic autoimmune diseases and infections, as well as purely ocular syndromes, can cause uveitis. Infectious agents were involved in the cross a etiology of uveitis in almost 19% of all cases. This high percentage was mainly due to the high numbers of ocular toxoplasmosis (7.0%) and herpetic uveitis (7.1%) patients. These numbers imply that correct classification can have a decisive impact on the success or failure of therapies, and that immunosuppressive medications should not be given without ruling out infectious a etiology. However, the diagnostic quest should not lead to generalized and extensive investigations that generate unnecessary costs. The recommended course is a tailored approach in the hands of uveitis specialists and/or rheumatologists/immunologists/infectiologists with uveitis experience. This would help to reinforce existing international guidelines, raise the standards of treatment, facilitate drug development, and shed light on the prognosis and course of the disease.

## Abbreviations

ARN: Acute retinal necrosis; APMPPE: Acute posterior multifocal placoid pigment epitheliopathy; AU: Anterior Uveitis; CMV: Cytomegaly virus infection; HCF: Fuchs’ heterochromic cyclitis; JIA: Juvenile idiopathic arthritis; MS: Multiple sclerosis; MASQ: Masquerade syndromes; SLE: Systemic lupus erythematosus; SUN: Standardization of Uveitis nomenclature; VKH: Vogt-Koyanagi-Harada disease; VZV: Varicella virus infection; WDS: White dot syndrome.

## Competing interests

The authors declare that they have no competing interests.

## Authors’ contributions

The work presented here was carried out in collaboration between all authors. TBA and SMM defined the research theme. LM, WE, KM and HA analyzed the data and interpreted the results. TBA wrote the paper. All authors have contributed to, seen and approved the manuscript.
